# Assessing the Relationship Between Religious Beliefs and Ethnicity and Handedness and Footedness

**DOI:** 10.7759/cureus.50688

**Published:** 2023-12-17

**Authors:** Taim Muayqil, Alhanouf Alhaluli, Lama Alzamil, Renad K AlKanaan, Yasmeen Almousa, Rana Alshamrani

**Affiliations:** 1 Neurology, Department of Internal Medicine, College of Medicine, King Saud University, Riyadh, SAU; 2 College of Medicine, King Saud University, Riyadh, SAU

**Keywords:** waterloo scale, cultural pressure, saudi arabia, muslim, arab, footedness, handedness

## Abstract

Objectives

The objective of the study is to explore the correlation between handedness and footedness and various demographic factors, including sex, age, faith, ethnicity, and perceived social pressures on limb use, among Muslims and non-Muslims.

Methods

This is an analytical cross-sectional study conducted in Saudi Arabia from September 2020 to February 2021. This research involved healthy community members aged 18 and above. An electronic survey was administered to collect demographic information on age, sex, faith, ethnicity, and the perceived degree of social pressure favoring the use of the right hand. The Waterloo handedness questionnaire (WHQ-Ar) and Waterloo footedness questionnaire (WFQ-Ar) were utilized for the assessments.

Results

A total of 728 respondents completed the survey. The mean (SD) age was 34.54 (11.53). Among them, 454 (62.4%) were ethnically Arab, and 507 (69.64%) identified as Muslims. The mean (SD) WHQ-Ar and WFQ-Ar scores were 42 (31.6) and 10.51 (8.1), respectively, with higher scores indicating “right” preference. Older age was associated with higher scores (p = 0.01) and men with lower handedness scores (p = 0.003). Participants who did not perceive social pressure had significantly lower scores (p < 0.001). Footedness was associated with slightly lower scores in Muslims (p = 0.001).

Conclusion

The degree of pressure perceived to use a particular hand has a major influence on handedness scores in Muslims and non-Muslims alike; this was true even when comparing populations with different levels of permissiveness to specific hand use due to varying religious or cultural backgrounds. Social pressure, regardless of routine religious practices, is more likely to influence handedness scores. Age and sex influence the scores similar to international reports, and footedness appears less likely to fall under the influence of cultural pressures.

## Introduction

Reports of handedness, in general, suggest that, on average, 90% of humans tend to depend on their right hand for skilled coordinated movements, such as writing, equipment use, and even bimanual activities, and both genetic and environmental factors play a role [1-4]. Handedness and footedness generally represent one’s preference to use an upper or lower limb, respectively, more often than the contralateral limb [1]. Handedness and footedness measures have been available for many decades [5-7]. Determination of cerebral lateralization of various cognitive functions is of major importance and frequently required prior to different interventions, for example, epilepsy surgery [8]. Scales for handedness are frequently used as part of more detailed neuropsychological assessments to infer the laterality of various cerebral functions [9].

While most individuals are right-handed across all societies, cultural pressures may have an influence on the expression of right-hand use [2,7,10]. Other biases such as that seen in line bisection tasks also vary across cultures [11]. Contrary to this, footedness was found to be less influenced by social pressure [6,12]. A study in the majority Muslim nation of Turkey found that younger generations were more left-handed than older individuals, which suggested that newer generations may be influenced by more permissive attitudes toward handedness [13]. Left-handedness rates, however, range from 5% to 25.9% in general, with men having higher rates than women [1,7]. The prevalence of a handedness type is also dependent to a degree on culture and region [1,2,5-7,12]. Differences in the preferred writing hand can vary up to 7% among cultures that exhibit different levels of applied formality [7]. While numerous previous studies have focused on investigating handedness more than footedness, footedness, however, may actually serve as a more precise predictor of functional laterality and motor abilities than handedness, as it is less likely to fall under the influence of cultural and environmental factors [14,15]. It has been argued that footedness should be a part of the neuro-psychological assessment because of the sensitivity of the feet in reflecting the functional characteristics of the motor system [16].

Differences according to sex, age, race, and faith have long been described in Western literature [17]. Social pressure, which is known to have a role in determining handedness, may not be purely related to religious obligations, but more of an embedded cultural aspect, as seen in Chinese samples or in societies where left-handedness is associated with a negative stigma [12,18]. Due to the lack of studies surrounding the topic of ethnic or cultural effects on handedness and footedness scores, specifically in the Arabian Peninsula, we aimed to determine which of the above-mentioned variables could potentially influence the degree of handedness and footedness, particularly with regard to faith, ethnicity, and perceived effects of social pressure, in addition to age and sex [2,5,7]. We assessed handedness and footedness with the Waterloo handedness questionnaire (WHQ-Ar) and Waterloo footedness questionnaire (WFQ-Ar) in a society with a known strong preference for the right and compared their results to individuals of non-Muslim faith or non-Arab ethnicity [10,13]. The latter group is assumed to be permissive with hand and foot preference in general. The questionnaires are composed of multiple Likert-type questions that inquire about preferred hand or foot use for various tasks. It is based on self-report, easy to administer, and commonly used in practice [6,12]. If the rightward tendencies and social pressures, to use a specific hand or foot, in the local society are enough to significantly change or skew the scores on such scales (yielding, for example, scores that suggest higher rates of right-handedness or footedness), this will then suggest that further exploration of the role of these scales in determining hemispheric lateralization in certain societies would be needed and that further in-depth neuropsychological assessment of an individual’s laterality may be required.

## Materials and methods

This project was a quantitative analytical cross-sectional study and was conducted between September 2020 and February 2021. An electronic survey was distributed to healthy participants at least 18 years of age of any ethnicity and faith who resided in Saudi Arabia.

At the time of selecting their preferred language (Arabic or English) and initiating the survey, participants provided consent, and data on handedness and footedness were collected through a self-administered electronic questionnaire, employing convenience sampling and the snowball method of recruitment. The WHQ-Ar and WFQ-Ar were used. The Waterloo scales are detailed scales of handedness and footedness and have excellent consistency with self-reported handedness (hand used for writing) and other shorter measures [7]. Both scales are well-established English tools for measuring the degree of handedness and footedness, and the recently validated Arabic versions are available, which show a strong correlation with self-described handedness and footedness [6,19-21]. The WHQ consists of 36 items that ask about the preferred hand use in various tasks. Similarly, the items of the WFQ-Revised explore which foot is chosen in performing a certain motor task. One of its main advantages versus other questionnaires of footedness is that it has been implemented to assess both foot preferences for bilateral mobilizing tasks, such as kicking a ball towards a target, and for unilateral stabilizing tasks as standing on one leg.

Participants' demographic data related to age, sex, faith (Muslim or non-Muslim), and self-described ethnicity (Arab or non-Arab) were assessed. Additionally, the degree of perceived social pressure toward using a particular hand and foot was assessed with two Likert-type questions, one for hand and the other for foot use. For example, "While growing up did your family or social environment encourage you to use one hand (foot) over the other?"; responses: "strongly agree, agree, neutral, disagree, strongly disagree." Moreover, sex, age, and religion were analyzed to determine their influence on handedness and footedness classification, given religion may be a factor related to social pressures.

The dominant hand and foot of the respondent are classified through the total score obtained in their answers to the Waterloo questionnaires. There are 36 and 10 items on the handedness and footedness scale, respectively; each item is scored on a Likert-type scale ranging from -2 (left always) to +2 (right always). The scores are then added up, with the overall handedness score falling between -72 and +72, and the overall footedness score from -20 to +20. This study was approved by the College of Medicine Institutional Review Board of King Saud University.

Descriptive statistics (mean, standard deviation (SD)) were used to describe quantitative data. While frequencies and percentages were used to describe qualitative data, T-tests and chi-square tests were carried out according to data type. A p-value of <0.05 and a 95% confidence interval were used to report the statistical significance and precision of the results. ANOVA was used to assess for differences in scores according to the degree of perceived social pressure. Spearman's method was used to test the correlation between variables in regard to strength and direction. Multiple regression was performed to estimate how the degree certain variables would contribute to changes in the final handedness and footedness scores. Data were analyzed using Stata software (version 15; StataCorp LLC, College Station, Texas).

## Results

The total number of participants in this study reached 1,066, and 728 completed the survey. The ages of the respondents ranged from 18 to 78 years old with an overall mean (SD) age of 34.54 (11.53). Mean (SD) 543 (74.59%) females and mean (SD) 185 (25.41%) males participated (Table 1). A total of 454 (62.4%) identified as ethnically Arab, and 507 (69.64%) identified as being Muslim. The mean (SD) handedness and footedness scores were 42 (31.6) and mean (SD) 10.51 (8.1), respectively. Variations in scores according to variables are demonstrated in Table 1. Overall, the proportion of those classified as right-handed according to score was 619 (85.03%), left-handed 53 (7.28%), and mixed-handed 56 (7.69%). Similarly, the proportion of those classified as right-footed was 491 (70.65%), left-footed 181 (26.04%), and mixed-footed 23 (3.31%).

**Table 1:** Baseline characteristics, scores, and distribution among variables. ^1 ^Total footedness scores available from 695 participants 515 females and 180 males.

	Sex		p-value	t-value	Faith		p-value	t-value	Ethnicity		p-value	t-value
	Male n = 185	Female n = 543			Muslim n = 507	Other n = 221			Arab n = 454	Non-Arab n = 274		
Age m (SD)	33.77 (11.25), range: 18-66	34.8 (11.6), range: 18-78	p = 0.3	t (726) = 1.04	34.5 (12.7), range: 18-78	34.63 (8.19), range: 18-60	p = 0.89	t (726) = -0.14	34.1 (12.75)	35.26 (9.11)	p = 0.19	t (726) = -1.32
Handedness score m (SD)	35.37 (38.38), range: from -72 to 72	44.22 (28.65), range: from -72 to 72	p = 0.0012	t (726) = 3.26	41.8 (27.28), range: from -67 to 72	42.44 (39.87), range: from -72 to 72	p = 0.8	t (726) = 0.25	40.85 (27.59), range: from -67 to 72	43.91 (37.3), range: from -72 to 72	p = 0.2	t (726) = -1.3
Right-handed, n (%)	144 (77.8%)	475 (87.5%)			434 (85.6%)	185 (83.71%)			387 (85.2%)	232 (84.7%)		
Mixed-handed, n (%)	20 (10.81%)	36 (6.63%)	X^2^ (2) = 10.3, p = 0.006		46 (9.07%)	10 (4.52%)	X^2^ (2) = 12.97, p = 0.002		42 (9.25%)	14 (5.1%)	X^2^ (2) = 9.03, p = 0.01	
Left-handed, n (%)	21 (11.35%)	32 (5.89%)			27 (5.33%)	26 (11.76%)			25 (5.51%)	28 (10.22%)		
	n = 180,	n = 515,			n = 479,	n = 216,			n = 426	n = 269		
Footedness score ^1^	9.27 (8.7), range: from -20 to 20	10.94 (7.8), range: from -20 to 20	p = 0.02	t (693) = 2.4	9.745 (7.6), range: from -18 to 20	12.19 (8.73), range: from -20 to 20	p = 0.0002	t (693) = 3.73	9.54 (7.58), range: from -18 to 20	12.03 (8.52), range: from -20 to 20	p = 0.0001	t (693) = -4
Right-footed, n (%)	121 (67.2%)	370 (71.8%)			324 (67.64%)	167 (77.3%)			283 (66.4%)	208 (77.32%)		
Mixed-footed, n (%)	48 (26.7%)	133 (25.8%)	X^2^ (2) = 6.2, p = 0.045		141 (29.4%)	40 (18.52%)	X^2^ (2) = 9.5, p = 0.009		130 (30.52%)	51 (18.96%)	X^2^ (2) = 11.45, p = 0.003	
Left-footed, n (%)	11 (61%)	12 (2.3%)			14 (2.92%)	9 (4.7%)			13 (3.05%)	10 (3.72%)		

On both the handedness and footedness scores, females had higher mean scores than males. The results of the perception of social pressure were completed by 356 participants and are presented in Table 2.

**Table 2:** Social pressure data.

Handedness		Sex		Faith		Ethnicity		Handedness score	
Experienced social pressure		Male	Female	Muslim	Other	Arab	Other	Mean (SD)	range
Strongly agree	90 (25.3%)	21 (32.8%)	69 (23.6%)	57 (42.2%)	33 (14.9%)	37 (43.5%)	53 (19.6%)	52.63 (22.42)	-56, 72
Agree	111 (31.2%)	13 (20.3%)	98 (33.6%)	39 (28.9%)	72 (32.6%)	28 (32.9%)	83 (30.6%)	45.11 (26.9)	-58, 72
Neutral	61 (17.1%)	14 (21.9%)	47 (16.1%)	18 (13.3%)	43 (19.5%)	9 (10.6%)	52 (19.2%)	50.03 (27.35)	-43, 72
Disagree	52 (14.6%)	5 (7.8%)	47 (16.1%)	17 (12.6%)	35 (15.8%)	9 (10.6%)	43 (15.9%)	40.5 (32.6)	-51, 72
Strongly disagree	42 (11.8%)	11 (17.2%)	31 (10.6%)	4 (2.9%)	38 (17.2%)	2 (2.4%)	40 (14.8%)	10.83 (62.7)	-72, 72
Totals	356	64	292	135	221	85	271		
P value			X^2^ (4) = 10.1, p = 0.04		X^2^ (4) = 41.88, p <0.001		X^2^ (4) = 27.29, p <0.001	Anova F(4) = 12.7, p	
Footedness		sex		Faith		Ethnicity		Footedness score	
Experienced social pressure		Male	Female	Muslim	Other	Arab	Other	Mean (SD)	range
Strongly agree	38 (10.7%)	12 (18.8%)	26 (8.9%)	25 (18.5%)	13 (5.9%)	10 (11.8%)	28 (10.3%)	13.4 (6)	-1, 20
Agree	54 (15.2%)	4 (6.3%)	50 (17.1%)	20 (14.8%)	34 (15.4%)	10 (11.8%)	44 (16.2%)	11.8 (8.2)	-20, 20
Neutral	100 (28.1%)	17 (26.6%)	83 (28.4%)	39 (28.9%)	61 (27.6%)	26 (30.6%)	74 (27.3%)	11.1 (8.6)	-18, 20
Disagree	97 (27.3%)	12 (18.8%)	85 (29.1%)	31 (23%)	66 (29.9%)	22 (25.9%)	75 (27.7%)	10.3 (8.6)	-17, 20
Strongly disagree	67 (18.8%)	19 (29.7%)	48 (16.4%)	20 (14.8%)	47 (21.3%)	17 (20%)	50 (18.5%)	11.7 (9)	-20, 20
Totals	356	64	292	135	221	85	271		
P value			X^2^ (4) =15.89, p = 0.003		X^2^ (4) = 15.9, p = 0.003		X^2^ (4) = 1.38, p = 0.847	Anova F (4) = 1.03, p = 0.3895	

The correlation between handedness and footedness scores was 0.6. There was a high correlation by Spearman’s method between being Muslim and of being Arab ethnicity (0.83), and only four individuals who were ethnically Arab identified themselves as non-Muslim. Considering the small number and concern for collinearity, the ethnicity variable was not included in the regression analysis. Additionally, to allow for larger sample sizes within the social pressure categories, respondents who chose “strongly agree” or “agree” to the effects of social pressure to use a particular limb were merged into a single “perceived” variable, and those who chose “disagree” or “strongly disagree” into a “non-perceived” variable. ANOVA showed a significant difference in the effect of the three-level social pressure perception variable on the handedness score (F (2, 353) = 14.02, p < 0.001), but not on the footedness score (F (2, 346) = 1.13, p = 0.33). We then checked if an interaction effect was present for being Muslim and the perception of pressure towards a specific hand and foot. The interaction was not significant between Muslim and social pressure on footedness score (F (5, 343) = 2.15, p = 0.06). However, an interaction was found during handedness score regression between being Muslim and the perception of pressure on hand use (F (5, 350), p < 0.0001). Therefore, the three-level social pressure variable and the interaction term were included in the handedness score regression, but not in the footedness score regression. In the final handedness regression, including the interaction variable, the interaction did not have a significant effect (Table 3). The post-handedness score regression mean VIF was 2.54 (ranging from 1.04 to 3.2), and the post-footedness score regression mean VIF was 1.03 (ranging from 1.0 to 1.04), both indicating the lack of multicollinearity.

**Table 3:** Multiple regression with the handedness score and footedness score as dependent variables. *indicates the interaction between being Muslim and the perception of social pressure.

	Handedness				Footedness			
	Coef. (SE)	p-value	t-value	95% CI	Coef. (SE)	p-value	t-value	95% CI
Age	0.45 (0.18)	0.01	2.52	0.1, 0.81	0.08 (0.026)	0.002	3.05	0.03, 0.13
Male	-14.39 (4.74)	0.003	-3.03	-23.7, -5.06	-1.16 (0.7)	0.1	-1.66	-2.53, 0.21
Muslim	-0.48 (4.7)	0.92	-0.1	-9.79, 8.83	-2.23 (0.66)	0.001	-3.37	-3.28, 1.18
Perceived social pressure								
Perceived	Ref.							
Neutral	3.56 (6.02)	0.55	0.59	-8.28, 15.4				
Did not perceive	-22.52 (5.1)	< 0.001	-4.42	-32.55, -12.5		-		
Interaction of “Muslim” with “perceived social pressure”								
Muslim*Perceived	Ref							
Muslim*Neutral	-4.68 (10.53)	0.66	-0.44	-25.39, 16.04				
Muslim*Did not perceive	10.09 (9.49)	0.29	1.06	-8.58, 28.76				

Multiple regression (Table 3) was performed to explore the effects of the variables age, sex, faith, Arab ethnicity, and social pressure on handedness score and the variables of variables age, sex, faith, and Arab ethnicity footedness scores. Social pressure on footedness use was not significant in the ANOVA (Table 2) and was not used in the multiple regression. Results are demonstrated in Table 3. There was no significant multicollinearity among the variables. VIF mean post-handedness score regression was 1.51, with the individual variable VIF values ranging from 1.08 to 1.93. VIF mean post-footedness score regression was 2.05, with individual variable values ranging from 1.01 to 3.08.

## Discussion

This study explored the influence of certain variables on handedness and footedness scores as measured by the Waterloo scales: age, gender, faith, and a perception of social pressure associated with effects on handedness and footedness scores to differing degrees.

Handedness and footedness distribution

The distribution of handedness classification in our group was close to that found in other regional studies. In studies from Turkey (a Muslim-majority country), right-handedness ranged from 79.15% to 93.3% [3,5,22]. Little research has been performed on handedness distribution in Saudi Arabia, and one survey aimed at identifying left-handedness in dental trainees found the left-handed frequency to be 6.6%, not far from, the frequency found here using a validated scale [23].

Regarding handedness classification based on the score obtained, although more Muslims were classified as right-handed, there was a non-significant difference. Mixed-handedness was found more often in Arabs and Muslims than left-handedness. This finding also suggests the effect of faith on handedness. Particularly since mixed-handedness in the non-Arab and non-Muslim groups was less than left-handedness. This difference in the final classification of an individual’s handedness requires further exploration, particularly to see if adhering to important daily practices such as eating with the right hand, with a permissive attitude toward other activities, would skew an individual’s score by a large enough degree to the mixed handedness group in an individual with left-handed tendencies.

Concerning the footedness classification, the distribution of participants within each group differed from the pattern seen in handedness. Percentages dropped from right to mixed and finally to left-footedness groups, regardless of faith or ethnicity. The correlation between handedness and footedness was similar to that obtained from a large Chinese cohort that found it to be 0.64 [12].

Age and sex

The variables sex and age were associated with handedness classification by the Waterloo scale pre-determined cutoffs. Our results have shown that sex affects the possibility of being left-handed and males had lower handedness scores than females. This difference by sex is well-documented, it has been proposed that laterality may be under hormonal influence [2,5,7,13,17,24,25]. Previous reports have found that some athletes, who were mixed or left-footed, reported more strength and endurance, possibly related to in-utero testosterone effects on cerebral lateralization. Sex differences are explained by biological reasons given the different effects of hormones on cerebral lateralization and brain plasticity more than the effects of social pressure on individual members of each sex [2,4,7,14,25].

About age, we observed that older participants were more likely to be right-handed than their younger counterparts. This is consistent with literature from various sources, as it has been proposed that laterality increases with increased experience and maturation [2]. This also aligns with the findings of a study from Turkey where siblings were more left-handed in comparison to parents [13].

Whereas in the footedness score, age similarly showed an association, with scores tending toward the positive. Specifically, each one-year increase in age was associated with a 0.08 positive increase in the footedness score. Initial results indicated that males had a higher likelihood of being left-footed than females, although this significance did not persist after the regression analysis.

Social pressure, faith, and ethnicity

Ethnic differences such as more left-handed tendencies occurring in Blacks compared to Whites and in Europeans compared to North African Arab populations have been described in the literature [10,17]. However, it was important to exclude ethnicity from further analysis in the current study, even if initial exploratory statistics suggested a difference, due to the imbalanced distribution of other variables. The handedness scores were not significantly associated with being Muslim after correction in multiple regression. The unexpected finding, however, was a significant effect of being Muslim on the footedness score, resulting in a more negative value. While this value was modest (-2.2 points for Muslims on average), this interesting finding warrants further exploration in large cohorts to determine if reproducible. Social pressure was not an important factor in footedness, and this can be due to the reasons explained below.

Among the main observations in this study is that social pressure had a significant association with handedness scores. Our study initially revealed that approximately 56.5% (both “agree” and “strongly agree” groups) experienced social pressure to use the right hand. The social pressure effect was significant in that the handedness score would be on average 22.5 points less in individuals who did not experience social pressure in comparison to those who did experience pressure. The effects on the handedness score are in line with previous studies that showed a significant presence of social pressure to use the right hand [7,12]. According to a study that compared Tunisians with French children, there were 7-14% more left-handed preferences for individual scale items in the French group, with differences being most prominent in the youngest age groups [10]. The study concluded that there is a role for cultural pressure, to even extend beyond Islam’s emphasis on eating only with the right hand. Similarly, cultures that exhibited features such as strict hierarchy and strict differentiation of gender roles had fewer left-handed individuals than cultures with a less formal structure [7]. Strong cultural pressure toward handedness exists in non-Islamic societies, such as China, where frequencies of left-handedness have reached below 1% in some samples. On the other hand, higher frequencies of left-handedness, similar to those in Western societies, have been observed in Chinese-American children [12,18].

The effect of social pressure on handedness is not unique to humans, and it has been suggested that social pressure even exerts an effect on the laterality of primates who live within social units or hierarchies [2]. In a Turkish study, differences in handedness were thought to be related to cultural effects as well, though this was different from the conclusion from another Turkish study, where the authors argued that handedness is biological, and is not formed by culture, based on the consistency of their results with other numbers reported by different countries [13,15]. However, the study was based on self-identification of hand preference, rather than a measure of handedness by a scale, which may explain the different results. The validation study by Alorainy found only a moderate correlation of 0.47 between the Arabic WHQ and self-identified handedness [20]. Generally speaking, it appears that it is not the practices of a particular faith per se, but rather the social pressure from members of this faith that promotes right-handedness. Different individuals within a particular faith will likely experience different levels of social pressure, and as demonstrated here, those who had prominent left-handed scores expressed low amounts of social pressure. The teachings of Islam strongly encourage using the right in general, with emphasis on certain tasks, most prominent examples include eating or drinking but do not have a similar emphasis on other tasks, such as writing [10]. While this may lead to more mixed handedness for individuals with left-handed tendencies, it also explains why the social pressure effect is more relevant than simply belonging to a specific faith that teaches right favoritism for specific practices only, as an influencer of handedness tendencies. In other words, it is likely a Muslim can adhere to using the right for certain religious obligations without resulting in a major change of handedness tendencies unless this individual was in a cultural environment that was not very permissive. In the predominantly Muslim nation of Tunis, more left-handed writing appeared when children entered school, as there were no constraints for the writing hand of choice [10]. The lack of interaction found in our study supports that social pressure is a different-handedness influencing phenomenon from simply adhering to the practices of a specific faith or belonging to an ethnic group, and assumptions about skewed scores of handedness should not automatically be made. Another finding that highlights this dichotomy is the lack of association of social pressure with the footedness score. In Islam, initiating tasks with the right foot such as entering a mosque, or putting on the right shoe before the left, is encouraged. However, these are not complex tasks, and so this religious habit may not be enough to influence footedness scores, and along with this, social pressure is expected to be lower for lower limb-related tasks.

## Conclusions

In summary, social pressures have a major influence on handedness scores in Muslims and non-Muslims residing in an Arabian Gulf country as measured by the WHQ-Ar. In addition to age and gender, this was true even when comparing populations with diverse levels of permissiveness to specific hand use due to varying religious or cultural backgrounds. Social pressure regardless of routine religious Islamic practices is more likely to influence handedness scores, but not footedness scores. This is in line with the previous literature that suggests footedness superiority in determining laterality. It is warranted not to use these scales in isolation when assessing brain lateralization and to consider adding other tools. These results will hopefully add to the understanding of laterality differences among adults and their influencing factors.

The lack of non-Muslims among the Arab group limited our ability to analyze whether the results were truly due to the cultural effects within Arabs beyond that expected for the religion. However, Islamic teachings are so deep-rooted in Arabian culture for the vast majority of Arabs, and truly discriminating against these effects may not be unattainable as spill-over of cultural practices from Muslim to non-Muslim Arabs is a possibility. This study was conducted on a sufficient number of participants to convey the significance of our variables. Nevertheless, it is beyond the scope of our study to generalize our results, as our findings were limited by the low number of left- and mixed-handed participants overall. The social pressure data were available for approximately half of the total sample but should still be a good number to estimate. Future studies that can include individuals of similar ethnicities but belonging to various faiths that differ in the emphasis of using the right hand or foot would help isolate separate effects.
