# Correction: Assessing the Relationship Between Religious Beliefs and Ethnicity and Handedness and Footedness

**DOI:** 10.7759/cureus.c149

**Published:** 2024-01-03

**Authors:** Taim Muayqil, Alhanouf Alhaluli, Lama Alzamil, Renad K AlKanaan, Yasmeen Almousa, Rana Alshamrani

**Affiliations:** 1 Neurology, Department of Internal Medicine, College of Medicine, King Saud University, Riyadh, SAU; 2 College of Medicine, King Saud University, Riyadh, SAU

This article has been corrected to accurately reflect the affiliations of the authors. The university name of each of the authors has been changed from King Saud University Medical City to King Saud University.

The authors deeply regret that these errors were not identified and addressed prior to publication.

 

